# Contraceptive Use and Method Preference among Women in Soweto, South Africa: The Influence of Expanding Access to HIV Care and Treatment Services

**DOI:** 10.1371/journal.pone.0013868

**Published:** 2010-11-05

**Authors:** Angela Kaida, Fatima Laher, Steffanie A. Strathdee, Deborah Money, Patricia A. Janssen, Robert S. Hogg, Glenda Gray

**Affiliations:** 1 Faculty of Health Sciences, Simon Fraser University, Burnaby, Canada; 2 Perinatal HIV Research Unit, University of the Witwatersrand, Soweto, South Africa; 3 Division of Global Public Health, School of Medicine, University of California San Diego, San Diego, California, United States of America; 4 Women's Health Research Institute, Vancouver, Canada; 5 Department of Obstetrics & Gynaecology, University of British Columbia (UBC), Vancouver, Canada; 6 School of Population and Public Health, University of British Columbia (UBC), Vancouver, Canada; 7 Child and Family Research Institute, Vancouver, Canada; 8 BC Centre for Excellence in HIV/AIDS, Vancouver, Canada; University of Cape Town, South Africa

## Abstract

**Objective:**

Preventing unintended pregnancy among HIV-positive women constitutes a critical and cost-effective approach to primary prevention of mother-to-child transmission of HIV and is a global public health priority for addressing the desperate state of maternal and child health in HIV hyper-endemic settings. We sought to investigate whether the prevalence of contraceptive use and method preferences varied by HIV status and receipt of highly active antiretroviral therapy (HAART) among women in Soweto, South Africa.

**Methods:**

We used survey data from 563 sexually active, non-pregnant women (18–44 years) recruited from the Perinatal HIV Research Unit in Soweto (May–December, 2007); 171 women were HIV-positive and receiving HAART (median duration of use = 31 months; IQR = 28, 33), 178 were HIV-positive and HAART-naïve, and 214 were HIV-negative. Medical record review was conducted to confirm HIV status and clinical variables. Logistic regression models estimated adjusted associations between HIV status, receipt of HAART, and contraceptive use.

**Results:**

Overall, 78% of women reported using contraception, with significant variation by HIV status: 86% of HAART users, 82% of HAART-naïve women, and 69% of HIV-negative women (p<0.0001). In adjusted models, compared with HIV-negative women, women receiving HAART were significantly more likely to use contraception while HAART-naïve women were non-significantly more likely (AOR: 2.40; 95% CI: 1.25, 4.62 and AOR: 1.59; 95% CI: 0.88, 2.85; respectively). Among HIV-positive women, HAART users were non-significantly more likely to use contraception compared with HAART-naïve women (AOR: 1.55; 95% CI: 0.84, 2.88). Similar patterns held for specific use of barrier (primarily male condoms), permanent, and dual protection contraceptive methods.

**Conclusion:**

Among HIV-positive women receiving HAART, the observed higher prevalence of contraceptive use overall and condoms in particular promises to yield fewer unintended pregnancies and reduced risks of vertical and sexual HIV transmission. These findings highlight the potential of integrated HIV and reproductive health services to positively impact maternal, partner, and child health.

## Introduction

Nearly 80% of the world's 15.5 million HIV-infected women live in sub-Saharan Africa, where heterosexual intercourse is the primary mode of HIV transmission [1]. Each year, these women experience over 1.4 million pregnancies [2], of which an estimated 50–84% are unintended [3], [4], [5]. Many of these pregnancies contribute to distressing adverse outcomes for women, children, and their families. Every year, nearly 350,000 infants are infected with HIV via mother-to-child transmission (MTCT) [1]. Maternal mortality, the world's worst health inequity, is exacerbated in the context of HIV [6], with recent reports indicating that maternal deaths have increased considerably in regions of high HIV prevalence [7]. In addition, across sub-Saharan Africa there are an estimated 8.9 million maternal orphans due to HIV-associated mortality [8].

Addressing the current desperate state of maternal and child health in sub-Saharan Africa is a global public health priority embodied in Millennium Development Goals (MDGs) 4 (reducing child mortality) and 5 (improving maternal and reproductive health) [9]. Central to achieving these goals is the prevention of unintended pregnancy through increasing access to and use of effective contraception [10].

Among women living with HIV infection, the provision of contraceptive services to prevent unintended pregnancy is also a critical [11] but largely neglected strategy to prevent mother-to-child-transmission (PMTCT) of the virus [12]. A recent cost-effectiveness study showed that a PMTCT strategy focused on increasing contraception among HIV-positive women could avert 29% more HIV-positive births than prophylactic nevirapine alone, at the same level of expenditure [13]. However, the prevailing under-emphasis of reproductive health within HIV programming remains evident in the numbers: unwanted fertility among women living with HIV is estimated to account for 25% of infant infections (nearly 90,000 MTCT incidents every year) and 20% of infant mortality [14].

The little that is known about the prevalence and types of contraceptive use among HIV-infected women in sub-Saharan Africa originates from studies conducted prior to the widespread availability of highly active antiretroviral therapy (HAART), the standard of HIV treatment [3], [15], [16], [17]. This is due, in part, to the recency of the population-level HAART scale-up effort in the region [18]. By increasing life expectancy [19], [20], [21], decreasing morbidity [20], [22], and reducing vertical [23] and sexual [24] transmission risks, expanding access to HAART is dramatically reducing the health risks and barriers to reproduction among HIV-affected individuals and couples. This emerging reality of HIV as a manageable chronic disease, with HIV-infected individuals anticipated to live well into (and past) their peak reproductive years, has highlighted the importance of assessing the potential behavioral and biological impacts of HAART on contraceptive use, safety, and efficacy [25], [26], [27], [28], [29], [30], [31], [32].

Given the HIV hyper-endemic context of reproduction in Soweto, South Africa [1], [33], the primary objective of this study was to assess the prevalence of contraceptive use and to determine whether contraceptive use varies according to HIV serostatus and use and duration of HAART among sexually active women aged 18–44 years. A secondary objective was to determine the types of contraceptive methods used (including barrier, hormonal, permanent, and dual protection methods) and whether contraceptive method preferences vary by HIV status and HAART use. This research was conducted within *Kaida et al's* conceptual framework of the potential impact of HAART on fertility in sub-Saharan Africa [26], where HAART use is hypothesized to reduce individuals' perceived risk of HIV transmission and disease progression, ease concerns about the risks of reproduction, and alter contraceptive use patterns. As such, we hypothesized that HIV-positive women receiving HAART would be less likely to use contraception compared with HIV-positive women not receiving HAART, with increasing duration of HAART use associated with incrementally lower contraceptive use. In addition, we hypothesized that contraceptive prevalence among HIV-positive women receiving HAART and HIV-negative women would be similar.

## Methods

### Study Setting

With 5.5 million HIV-infected residents, South Africa is the country with the world's largest absolute number of people living with HIV [1]. The national adult (aged 15–49 years) HIV prevalence is 19% and the antenatal HIV prevalence is 30% [1]. The South African epidemic is highly feminized as women account for 60% of all infected adults and among 15–24 year olds, women account for 90% of incident HIV infections [34]. HIV prevalence in the study site, Soweto, an urban township of Johannesburg, is among the highest in the country [33].

Each year an estimated 220,000 women living with HIV in South Africa become pregnant [2]. Although coverage of prophylactic antiretroviral therapy to prevent MTCT increased from 15% to 73% between 2004 and 2008 [2], recent estimates report that over 64,000 infants are infected with HIV via MTCT each year [35]. Combination antiretroviral therapy became available in South Africa's public sector clinics in 2004 and by the end of 2008 an estimated 700 000 adults were receiving HAART, an antiretroviral therapy coverage of 31% [2].

Effective contraceptive methods, including injectables (*Nur-Isterate* and *Depo Provera*), oral contraceptive pills, the intrauterine device (IUD), condoms, and male and female sterilization, are available at no-cost in government health centres throughout Soweto. Elective termination of pregnancy is legal in South Africa and available at no-cost up to 12 weeks gestation.

This study was conducted at the Perinatal HIV Research Unit (PHRU), a large clinical and research site housed within one of the world's largest hospitals: Chris Hani Baragwanath Hospital in Soweto. The PHRU sees over 5 000 adult visits monthly and provides antiretroviral therapy and clinical care to medically-eligible HIV-positive individuals and ongoing wellness care for those not yet eligible for HAART. The PHRU also operates a Voluntary Counseling and Testing (VCT) centre. All PHRU services (including HIV prevention, testing, treatment and care and family planning) are provided free-of-charge. Since April 2006, onsite no-cost family planning services have been available to HIV-positive women accessing antiretroviral therapy at the PHRU. These services offer barrier, oral, and injectable contraceptive methods as well as family planning counseling from a trained family planning nurse. Treatment-naïve HIV-positive women may also access family planning services from the PHRU, however, they are not routinely queried about their contraceptive use during regular clinical follow-up. The PHRU primarily serves residents of Soweto.

### Study design

This analysis is based on cross-sectional survey data of HIV-positive (HAART receiving and HAART-naïve) and HIV-negative women seeking services at the PHRU. A medical chart review was also conducted to confirm HIV serology and HAART use history of HIV-positive women.

### Eligibility criteria

To be eligible to participate in the overall study, women were required to be 18–49 years of age, attending a PHRU clinic, residing in Soweto, competent to give informed consent, and willing to allow medical record review for the purposes of confirming HIV status and HAART history. We considered women to be HAART users if they had been receiving HAART for at least one month. We considered women to be HAART-naïve if they had never taken HAART.

### Study Sample

We enrolled 751 women, including 253 HIV-positive women receiving HAART, 249 HIV-positive but HAART-naïve women, and 249 HIV-negative women. This sampling strategy provided one case group (HAART users) and two comparison groups (HAART-naïve and HIV-negative women).

HAART users were sampled from the PHRU's President's Emergency Plan for AIDS Relief (PEPFAR) Clinic, which has provided free antiretroviral therapy to medically eligible patients since July 2004. Currently, the PEPFAR clinic has over 1,000 patients receiving HAART, 75% of who are female. PEPFAR patients are followed-up every three months and generally receive one of two standard HAART regimens. Regimen 1 consists of stavudine (d4T), lamivudine (3TC), and efavirenz (EFV) or nevirapine (NVP). Regimen 2 consists of zidovudine (AZT), didanosine (ddI), and lopinavir/ritonavir (LPV/r) [36].

HIV-positive HAART-naïve women were sampled from the PHRU's Wellness Clinic, initiated in January 2003 with the goal of providing preventive care to HIV-positive individuals. Wellness patients are followed-up approximately every six months. When patients become medically eligible for HAART, they are referred to the PEPFAR clinic or to one of the nearby government ART clinics. There are approximately 3,000 active patients in the Wellness Clinic.

HIV-negative women were sampled from the PHRU's VCT clinic, which was initiated in mid-2002 and sees approximately 400 people per month. Testing is conducted onsite during visits that last an average of two hours. Approximately 65% of attendees are women and 30% of all attendees test HIV-positive.

For this analysis on contraceptive use, we restricted the study sample to women aged 18–44 years who were currently sexually active (i.e., reported sexual activity in the previous six months), not currently pregnant, and pre-menopausal as per self-report). This was done to enhance the comparability of findings with other studies investigating reproductive and sexual health among HIV-positive populations. The restriction yielded an analytic sample of 563 women (75% of total sample), including 171 women on HAART, 178 HAART-naïve women, and 214 HIV-negative women.

### Data Collection

Every female patient attending the PEPFAR Clinic and the VCT clinic was consecutively approached by a research assistant to assess eligibility and interest in participating in the study. Since many more women attend the Wellness Clinic, a list was made of chart numbers of women attending the clinic each day. A random sample of chart numbers (40% of the total number of charts present) was then drawn and the corresponding women were approached to assess eligibility and interest in participating in the study.

After confirming eligibility and seeking written informed consent, all participants were asked to complete a 15–25 minute interviewer-administered questionnaire in English. The study interviewers were multilingual and trained to ensure accurate and consistent translation of the questionnaire if required or requested by the participant. Pilot testing of 45 women revealed that women were able to understand and answer the questionnaire. Pilot testing helped to ensure a more comprehensive list of responses were available for structured questions and that terms such as “sexual intercourse” were defined to refer specifically to “vaginal sex between a man and a woman”. Approximately 12 women were interviewed daily by three trained research assistants between May and December 2007. Research assistants were women from the local community who had previous research experience and were recent Social Sciences' graduates of a local university. Interviewers were supervised by an experienced research nurse. Two research nurses with HIV training conducted the medical record review. Participants were given transport reimbursement as compensation.

### Data Collection Instruments

The questionnaire assessed socio-demographic characteristics; HIV status, diagnosis, and treatment; clinical stage of disease; HAART history; fertility intentions; fertility history; contraceptive practices; and sexual history. The survey instrument was developed from a validated questionnaire used in an earlier pilot study [37].

We reviewed medical records of HIV-positive women to confirm HIV status and HAART history, and to obtain clinical data including CD4 cell counts and WHO stage of disease. Viral load measures were only available for women receiving HAART as only patients receiving treatment in the PEPFAR clinic undergo viral load testing.

### Measures

The primary outcome was self-reported contraceptive use in the previous six months. Contraceptive methods queried included male and female condoms (restricted to those reporting “Always” use), injections (*depomedroxyprogesterone acetate* (DMPA) or *norethisterone enantate*), oral contraceptive pills, diaphragm, intrauterine devices (IUD), female tubal ligation, hysterectomy, and male partner sterilization. In assessing the contraceptive method profile, dual protection was defined as use of both a barrier contraceptive method (primarily the male condom) and use of a hormonal or permanent contraceptive method [38].

The primary explanatory variables were HIV status and current receipt of HAART. Covariates included age, education, employment, household income, current sexual partnership, HIV status of regular sexual partners, parity, number of living children, fertility intentions, and HIV clinical variables.

### Statistical Analysis

We computed and compared the prevalence of contraceptive use between HIV-positive women and HIV-negative women overall and then between each of the three groups of women. We conducted two separate models to measure the presence and strength of the association between HAART use and the odds of contraceptive use, controlling for covariates. The first model compared HAART users and HAART-naïve women to HIV-negative women. The second model compared HAART users to HAART-naïve women and allowed adjustment for HIV-associated clinical characteristics.

In both models, univariate analyses were used to assess the relationship between HIV status, receipt of HAART, contraceptive use, and covariates. Differences in contraceptive use between groups are reported using Pearson's chi-squared test (for categorical variables) and ANOVA, or Student's independent t-test (for continuous variables). After testing for co-linearity (using Spearman's rho (*ρ*)) [39] and interaction [40], all covariates with significant associations in the univariate analysis were included in multivariate logistic regression models to obtain adjusted estimates of the association between HIV status, receipt of HAART, and contraceptive use. Age was forced into both multivariate models regardless of its univariate associations. All statistical tests were two-sided and considered significant at α = 0.05.

Among women who reported using contraception, we analyzed types of methods used by women in each of the three groups and overall. In addition to reporting use of each contraceptive method individually, we collapsed the data into four mutually exclusive groups including “Use of dual protection”, “Consistent condom use only”, “Use of Hormonal/Permanent method only”, and “Not using any contraceptive method” and tested for differences by HIV and HAART use status using Pearson's chi-squared test.

We assessed the association between duration of HAART use and prevalence of contraceptive use using Pearson's test for trend. HAART-naïve women were included in the “0 months on HAART” category.

### Sub-Analyses

We conducted the same analyses described above but restricted our sample to women aged 18–34 years to investigate the potential impact of differences in mean baseline age between HIV-positive and HIV-negative women in our study. This age group corresponds with the peak childbearing years among women in South Africa [41].

### Ethics Statement

All participants provided voluntary informed consent and all procedures were approved by the Human Research Ethics Committee of the University of the Witwatersrand, the University of British Columbia Health Research Ethics Board, the Simon Fraser University Office of Research Ethics, and the University of California San Diego Institutional Review Board. Information letters and consent forms were available in English and two local languages (isi-Zulu and Sesotho) to ensure comprehensive understanding of the study objectives, potential risks, and benefits.

## Results

Of 801 women approached for participation, 751 consented, completed the questionnaire, and underwent medical record review (participation rate = 94%). This analysis was restricted to 563 sexually active, non-pregnant women aged 18–44 years.

### Baseline characteristics

As shown in **Table 1**, there were differences in baseline characteristics by HIV and HAART use status. Mean age was 30 years [SD = 6.7], however, HIV-negative women were significantly younger than HIV-positive women. Half of the women had less than a grade 12 education, 62% were unemployed, and 71% had a monthly household income less than 3,000 ZAR ($380 USD). Nearly one-quarter (23%) reported that her primary sexual partner was HIV-positive, 29% reported that he was HIV-negative, and 42% did not know her partner's HIV status. Mean parity was 1.5 [SD = 1.2] and 44% of women had two or more living children. Nearly half (45%) reported intent to have more children.

**Table 1:** Baseline characteristics of HIV-positive (HAART users and HAART-naïve) and HIV-negative women (aged 18–44 years, currently sexually active and non-pregnant) in Soweto, South Africa (n = 563).

Variable	HAART users (n = 171)n (%)	HAART-naïve (n = 178)n (%)	HIV-negative (n = 214)n (%)	Overall (n = 563)n (%)	p-value¥
Mean Age (yrs) [SD]	33.7 [5.0]	32.3 [5.6]	25.3 [6.0]	30.0 [6.7]	<0.0001
Age Group (yrs)					<0.0001
18–24	4 (2%)	15 (8%)	119 (56%)	138 (25%)	
25–29	30 (18%)	41 (23%)	43 (20%)	114 (20%)	
30–34	66 (39%)	63 (36%)	37 (17%)	166 (30%)	
35–39	43 (25%)	35 (20%)	7 (3%)	85 (15%)	
40–44	27 (16%)	23 (13%)	8 (4%)	58 (10%)	
Education					<0.0001
Less than Grade 12	114 (67%)	109 (61%)	60 (28%)	283 (50%)	
Grade 12 or higher	56 (33%)	69 (39%)	154 (72%)	279 (50%)	
Employment Status					0.0962
Employed	71 (42%)	75 (42%)	70 (33%)	216 (38%)	
Unemployed	100 (58%)	103 (58%)	144 (67%)	347 (62%)	
Household income (per month)					<0.0001
Less than 3000 ZAR	142 (83%)	146 (82%)	111 (52%)	399 (71%)	
3,000 or more ZAR	16 (9%)	24 (13%)	65 (30%)	105 (19%)	
Don't know/Refused	13 (8%)	8 (4%)	38 (18%)	59 (10%)	
Currently in a sexual relationship					0.6337
No	12 (7%)	16 (9%)	14 (7%)	42 (7%)	
Yes	159 (93%)	162 (91%)	200 (93%)	521 (93%)	
HIV status of regular sexual partner/husband					<0.0001
Don't Know	64 (37%)	87 (49%)	83 (39%)	234 (42%)	
HIV-negative	30 (18%)	22 (12%)	111 (52%)	163 (29%)	
HIV-positive	69 (40%)	59 (33%)	2 (1%)	130 (23%)	
Single	8 (5%)	10 (6%)	18 (8%)	36 (6%)	
Mean parity [SD]	1.9 [1.1]	1.9 [1.2]	0.85 [0.9]	1.5 [1.2]	<0.0001
Number of living children					<0.0001
0	19 (11%)	19 (11%)	96 (45%)	134 (24%)	
1	58 (34%)	56 (31%)	68 (32%)	182 (32%)	
2 or more	94 (55%)	103 (58%)	50 (23%)	247 (44%)	
Fertility Intentions					
Yes	55 (32%)	55 (31%)	146 (68%)	256 (45%)	<0.0001
No	116 (68%)	123 (69%)	68 (32%)	307 (55%)	
Mean # of months since HIV diagnosis [SD]	69.0 [36.3]	50.8 [32.1]	N/A	59.7 [35.4]	<0.0001
Mean recent CD4 [SD]	405.7 [211.2]	349.4 [202.3]	N/A	376.8 [208]	0.0117
Mean nadir CD4 [SD]	110.1 [98.9]	309.4 [157.6]	N/A	212.7 [166]	<0.0001
WHO Stage of Disease					0.5267
Stage I/II	165 (98%)	173 (97%)	N/A	338 (98%)	
Stage III/IV	3 (2%)	5 (3%)		8 (2%)	
Disclosed HIV status to anybody					0.0493
No	4 (2%)	12 (7%)	N/A	16 (5%)	
Yes	167 (98%)	166 (93%)		333 (95%)	

Notes:

¥ Differences between groups are reported using Pearson's chi-squared test statistic (for categorical variables) and Student's independent t-test or ANOVA (for continuous variables); SD = Standard Deviation; N/A = Not Applicable.

Among HIV-positive women (n = 349), mean time since first HIV diagnosis was 59.7 months [SD = 35.4]. Half had recent CD4 counts ≥350 cells/mm^3^ (mean recent CD4 = 376.8 [SD = 208]) and 12% had nadir CD4 counts <50 cells/mm^3^ (mean nadir CD4 = 212.7 [SD = 166]). Nearly all women were in WHO Stage of Disease I or II (98%) and 95% had disclosed their HIV status to someone.

Among HAART users (n = 171), median duration of HAART use was 31 months [IQR: 28, 33], ranging from one to 89 months. Eighty percent of HAART users with recorded viral load measures were virally suppressed (<50 copies/ml).

### Prevalence of contraceptive use

Overall contraceptive prevalence was 78%. This varied significantly by HIV status with 84% of HIV-positive women (including 86% of HAART users and 82% of HAART-naïve women) and 69% of HIV-negative women reporting current contraceptive use (p<0.0001).

### Types of contraceptive methods used

Contraceptive method preferences are shown in **Table 2**. Part (a) of Table 2 shows mutually exclusive groups of contraceptive users. As shown, HIV-positive women overall were significantly more likely to use dual protection compared with HIV-negative women (33% and 14%, respectively). Much of this difference was accounted for by HAART users, of whom 40% reported using dual protection compared with 24% of HAART-naïve women and 14% of HIV-negative women. HAART users were also significantly more likely to report using condoms (with or without hormonal/permanent methods) (68%) and hormonal/permanent methods (with or without condoms) (58%) compared with HAART-naïve and HIV-negative women (p<0.0001).

**Table 2:** Types of contraceptive methods used by HIV-positive (HAART users and HAART-naïve) and HIV-negative women (aged 18-44 years, currently sexually active and non-pregnant) in Soweto, South Africa.

	HIV-positive women			HIV-negative women (n = 214)n (%)	Overall (n = 563)n (%)	p-value§
	HAART users (n = 171)n (%)	HAART-naïve women (n = 178)n (%)	All HIV-positive women (n = 349)n (%)			
**Contraceptive Prevalence**	**86%**	**82%**	**84%**	**69%**	**78%**	**<0.0001**
**(a) Mutually exclusive categories of type of contraceptive method used:**						**<0.0001**
Dual protection (Hormonal/permanent method AND consistent condom use)	40%	24%	33%	14%	25%	
Hormonal/Permanent method only	18%	23%	20%	30%	24%	
Consistent condom use only	28%	35%	31%	25%	29%	
Not using any contraceptive method	14%	18%	16%	31%	22%	

(b) Method use among contraceptive users (n = 411)*:	HIV-positive women			HIV-negative women (n = 148)n (%)	Overall (n = 441)n (%)	p-value§
	HAART users (n = 146)n (%)	HAART-naïve women (n = 147)n (%)	All HIV-positive women (n = 293)n (%)			
**Hormonal ** **Methods**	**88 (60%)**	**68 (47%)**	**156 (53%)**	**89 (60%)**	**245 (56%)**	**0.0211**
Injections	82 (56%)	49 (34%)	131 (45%)	66 (45%)	197 (45%)	0.0007
Oral Contraceptive Pill	6 (4%)	19 (13%)	25 (9%)	23 (16%)	48 (11%)	0.0041
**Barrier ** **Methods**	**116 (79%)**	**105 (72%)**	**221 (75%)**	**85 (57%)**	**306 (69%)**	**0.0001**
Consistent male condom use	116 (79%)	105 (72%)	221 (75%)	84 (57%)	305 (69%)	0.0001
Diaphragm	0 (0%)	0 (0%)	0 (0%)	1 (1%)	1 (<1%)	n/a
**Permanent methods**	**12 (8%)**	**20 (14%)**	**32 (11%)**	**4 (3%)**	**33 (7%)**	**0.0140**
Hysterectomy	4 (3%)	5 (3%)	9 (3%)	0 (0%)	9 (2%)	0.0897
Female sterilization	8 (5%)	15 (10%)	23 (8%)	4 (3%)	27 (6%)	0.0234

Notes:

§ p-value from chi-squared test statistics comparing proportions across three groups: HAART-users, HAART-naïve, and HIV-negative women;

* Values may not total 100% because one woman may report using more than one method.

Of the 411 women reporting contraceptive use (**Table 2 part (b)**), 56% used hormonal contraception, 69% used barrier methods (mainly the male condom), and 7% used permanent methods (i.e., hysterectomy and/or female sterilization) with significant differences by HIV status and HAART use. Across all three groups, hormonal contraceptive users utilised injectables more commonly than oral contraceptives. HAART users were significantly more likely to use condoms (79%), compared with HAART-naïve (72%) and HIV-negative (57%) women (p = 0.0001). Higher proportions of HIV-positive women had a tubal ligation or hysterectomy compared with HIV-negative women (p = 0.014).

### Univariate and Adjusted Analysis of Contraceptive use: HAART users, HAART non-users, and HIV-negative women

In the unadjusted analyses, many of the measured baseline covariates were significantly associated with contraceptive use (**Table 3**). Compared with HIV-negative women, HIV positive women were significantly more likely to use contraception (HAART users OR: 2.73; 95% CI: 1.62, 4.59; and HAART-naïve women OR: 2.04; 95% CI: 1.26, 3.29).

**Table 3:** Univariate and adjusted analyses of variables associated with contraceptive use among HIV-positive (HAART users and HAART-naïve) and HIV-negative women (aged 18–44 years, currently sexually active and non-pregnant) in Soweto, South Africa (n = 563).

Variable	Contraceptive Use		Crude OR		Adjusted OR	
	No (%)(n = 122)	Yes (%)(n = 441)	OR	95% CI	AOR	95% CI
**HIV and HAART Use Status**						
HIV-negative	66 (54%)	148 (34%)	Ref.	Ref.	**Ref.**	**Ref.**
HIV-positive, HAART-naïve	32 (26%)	146 (33%)	2.04	1.26, 3.29	**1.59**	**0.88, 2.85**
HIV-positive, receiving HAART	24 (20%)	147 (33%)	2.73	1.62, 4.59	**2.40**	**1.25, 4.62**
**Age** (per increase in year)	29.0 [SD = 7.4]	30.3 [SD = 6.5]	1.03	1.00, 1.06	**0.94**	**0.90, 0.98**
**Education**						
Less than Grade 12	45 (37%)	238 (54%)	Ref.	Ref.	Ref.	Ref.
Grade 12 or higher	77 (63%)	202 (46%)	0.50	0.33, 0.75	0.70	0.44, 1.13
**Employment Status**						
Unemployed	67 (55%)	280 (63%)	Ref.	Ref.	–	–
Employed	55 (45%)	161 (37%)	0.70	0.47, 1.05		
**Household income (per month)**						
Less than 3000 ZAR	72 (59%)	327 (74%)	Ref.	Ref.	Ref.	Ref.
3,000 or more ZAR	33 (27%)	72 (16%)	0.48	0.30. 0.78	0.94	0.55, 1.63
DK/Refused	17 (14%)	42 (10%)	0.54	0.29, 1.01	0.73	0.37, 1.43
**Currently in a sexual relationship**						
No	12 (10%)	30 (7%)	Ref.	Ref.	–	–
Yes	110 (90%)	411 (93%)	1.50	0.74, 3.02		
**HIV status of regular sexual partner/husband**					**–**	**–**
Don't Know	50 (41%)	184 (42%)	Ref.	Ref.		
HIV-negative	42 (34%)	121 (27%)	0.78	0.49, 1.25		
HIV-positive	22 (18%)	108 (25%)	1.33	0.77, 2.32		
Single	8 (7%)	28 (6%)	0.95	0.41, 2.22		
**Number of living children**						
0	43 (35%)	91 (21%)	Ref.	Ref.	**Ref.**	**Ref.**
1	50 (41%)	132 (30%)	1.25	0.77, 2.03	**1.01**	**0.59, 1.73**
2+	29 (24%)	218 (49%)	3.55	2.09, 6.04	**2.39**	**1.17, 4.89**
**Fertility Intentions**						
Yes	81 (66%)	175 (40%)	Ref.	Ref.	**Ref.**	**Ref.**
No	41 (34%)	266 (60%)	3.03	1.96, 4.55	**1.96**	**1.17, 3.29**

Notes:

Ref. = Reference category.

SD = Standard Deviation.

In adjusted analyses, compared with HIV-negative women, HAART users remained significantly more likely to use contraception (OR: 2.40; 95% CI: 1.25, 4.62) while non-HAART users were non-significantly more likely to use contraception (OR: 1.59; 95% CI: 0.88, 2.85). Overall, HIV-positive women (combining HAART users and HAART-naïve women) had significantly increased adjusted odds of 1.75 (95% CI: 1.02, 2.99) of using contraception compared with HIV-negative women. Younger age, having two or more living children, and expressing an intention not to have (more) children also remained significantly associated with contraceptive use.

### Univariate and Adjusted Analysis: HIV-positive women

In the analyses restricted to HIV-positive women (**table 4**), the unadjusted odds of reporting contraceptive use among HAART users and HAART-naïve women were not significantly different (OR: 1.34; 95% CI: 0.75, 2.39). There were no significant differences in contraceptive use by HIV clinical characteristics.

**Table 4:** Univariate and adjusted analyses of variables associated with contraceptive use among HIV-positive women (aged 18–44 years, currently sexually active and non-pregnant) in Soweto, South Africa (n = 349).

Variable	Contraceptive Use		Crude OR		Adjusted OR	
	No (%)(n = 56)	Yes (%)(n = 293)	OR	95% CI	OR	95% CI
**HAART Use**						
HIV-positive, HAART-naïve	32 (57%)	146 (50%)	Ref.	Ref.	Ref.	Ref.
HIV-positive, receiving HAART	24 (43%)	147 (50%)	1.34	0.75, 2.39	1.55	0.84, 2.88
**Age** (per increase in year)	33.3 [SD = 5.6]	32.9 [SD = 5.3]	0.99	0.94, 1.04	**0.93**	**0.88 0.99**
**Education**						
Less than Grade 12	29 (52%)	194 (66%)	Ref.	Ref.	Ref.	Ref.
Grade 12 or higher	27 (48%)	98 (34%)	0.54	0.30, 0.97	0.62	0.33, 1.17
**Employment Status**						
Unemployed	31 (55%)	172 (59%)	Ref.	Ref.	–	–
Employed	25 (45%)	121 (41%)	0.87	0.49, 1.56		
**Household income (per month)**						
Less than 3000 ZAR	44 (79%)	244 (83%)	Ref.	Ref.	–	–
3,000 or more ZAR	8 (14%)	32 (11%)	0.72	0.31, 1.67		
DK/Refused	4 (7%)	17 (6%)	0.77	0.25, 2.39		
**Currently in a sexual relationship**						
No	5 (9%)	23 (8%)	Ref.	Ref.	–	–
Yes	51 (91%)	270 (92%)	1.15	0.42, 3.17		
**HIV status of regular sexual partner/husband**					–	–
Don't Know	25 (45%)	126 (43%)	Ref.	Ref.		
HIV-negative	8 (14%)	44 (15%)	1.09	0.46, 2.60		
HIV-positive	21 (38%)	107 (37%)	1.01	0.54, 1.91		
Single	2 (4%)	16 (5%)	1.59	0.34, 7.34		
**Number of living children**						
0	11 (20%)	27 (9%)	Ref.	Ref.	**Ref.**	**Ref.**
1	27 (48%)	87 (30%)	1.31	0.58, 2.99	**1.12**	**0.47, 2.65**
2+	18 (32%)	179 (61%)	4.05	1.73, 9.50	**3.07**	**1.18, 7.96**
**Fertility Intentions**						
Yes	30 (54%)	80 (27%)	Ref.	Ref.	**Ref.**	**Ref.**
No	26 (46%)	213 (73%)	3.03	1.72, 5.56	**2.22**	**1.15, 4.35**
**Mean # of months since HIV diagnosis [SD]**	59.6 [SD = 33.1]	59.8 [SD = 35.9]	1.00	0.99, 1.01	–	–
**Recent CD4**						
<200	8 (14%)	54 (19%)	Ref.	Ref.	–	–
200 to <350	22 (39%)	88 (30%)	0.59	0.25, 1.43		
350 or greater	26 (46%)	148 (51%)	0.84	0.36, 1.97		
**Nadir CD4**						
<50	7 (13%)	36 (12%)	Ref.	Ref.	–	–
50 to <200	22 (39%)	133 (46%)	1.18	0.47, 2.97		
200 to <350	15 (27%)	63 (22%)	0.82	0.31, 2.19		
350 or greater	12 (21%)	58 (20%)	0.94	0.34, 2.61		
**WHO Stage of Disease**						
Stage I/II	55 (98%)	283 (98%)	Ref.	Ref.	–	–
Stage III/IV	1 (2%)	7 (2%)	1.36	0.17, 11.3		
**Disclosed HIV status to anybody**						
No	0 (0%)	16 (5%)	N/A	N/A	–	–
Yes	56 (100%)	277 (95%)				

Notes:

Ref. = Reference category; SD = Standard Deviation; N/A = Not applicable.

In adjusted analyses, HAART users were more likely than non-users to report contraceptive use, however the difference was not statistically significant (AOR: 1.55; 95% CI: 0.84, 2.88). Younger age, having two or more living children, and expressing an intention not to have more children remained most strongly associated with contraceptive use.

### Contraceptive use by duration of HAART use

**Figure 1:**
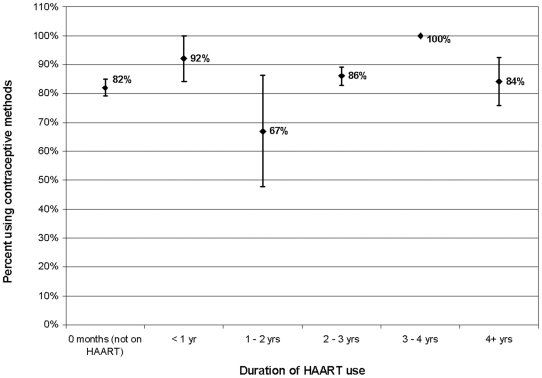
Percentage of HIV-positive women using contraception, by length of time on highly active antiretroviral therapy (HAART): Soweto, South Africa (n = 349).

There was no clear association between duration of HAART use and prevalence of contraceptive use. The prevalence of contraceptive use remained steady (between 82% and 92%) for all lengths of time on HAART with the exception of women receiving HAART between one and two years, who had the lowest prevalence of contraceptive use at 67% (**Figure 1**). However, as there were few women receiving HAART for 1–2 years, the 95% confidence interval on this estimate is very wide.

### Sub-analyses

Contraceptive prevalence of young women (18–34 yrs, n = 420) was similar to the overall sample and still varied significantly by HIV and HAART use status: 88% of HAART users, 82% of HAART-naïve women, and 70% of HIV-negative young women reported using contraception (p = 0.0007). In multivariate analyses we found that the same variables associated with contraceptive use in the overall sample were similarly associated with contraceptive use among young women. Compared with HIV-negative women, HAART users remained significantly more likely to use contraception (AOR: 2.24; 95% CI: 1.05, 4.99) while HAART-naïve women were similarly likely to use contraception (AOR: 1.27, 95% CI: 0.65, 2.48) (data shown in Table S1).

## Discussion

We found that HIV-positive women overall were significantly more likely to use contraception compared with HIV-negative women. In particular, and in contrast with our hypothesis, women receiving HAART were significantly more likely to report contraceptive use while HIV-positive HAART-naïve women were non-significantly more likely to use contraception compared with HIV-negative women. Overall, over 80% of HIV-positive women in our study reported contraceptive use, which falls within the upper range reported for HIV-positive women elsewhere in sub-Saharan Africa (46%–85%) [16], [17], [25], [32], [42], [43]. Contraceptive prevalence among HIV-negative women in our study was 69%, which is highly comparable to estimates among women in the general South African population [41].

Among HIV-positive women, our finding indicating non-significantly higher prevalence of contraceptive use among HAART users compared with HAART-naïve women is broadly consistent with recent findings from Uganda [25]. Other factors associated with contraceptive use included younger age, having two or more living children, and a lack of intention to have more children, all of which are widely reported to influence contraceptive decision-making [17], [25], [44]. To the best of our knowledge, this is the first study to show no significant change in prevalence of contraceptive use by duration of HAART use.

The reasons for higher contraceptive prevalence among HIV-positive women in general and HAART users in particular were not directly explored in this study. However, an important possible reason for the observed differences is that women receiving HIV treatment and care have more regular contact with health care professionals as a function of the clinical follow-up required to monitor the health of HIV-positive individuals. During these regular clinic visits, reproductive and sexual health issues are raised and the opportunity to discuss and commence use of contraception is presented. Among HIV-positive women receiving HAART at the PHRU in particular, discussions about family planning are incorporated into the regular clinical follow-up routine of PEPFAR patients. Moreover, although contraceptive methods are freely available at numerous public health sector sites in Soweto, it is likely that HIV-positive women are benefitting from longitudinal and integrated regular contact with the PHRU (a non-governmental health care facility) versus the intermittent contact with government clinics more commonly experienced by women not receiving HIV treatment and care [41]. It may also be likely that HIV-positive women opting and/or able to receive HIV treatment and care may have higher levels of self-empowerment than those not in care, which may contribute to higher rates of contraceptive use [45]. Additional studies are required to explore and determine the specific pathways that support higher contraceptive use among women accessing HAART in this setting.

The empirical findings reported here are inconsistent with the hypothesized effects of HAART described in our conceptual framework [26]. It must be noted that this conceptual framework was developed by reviewing literature from the very early days of HAART scale-up initiatives in sub-Saharan Africa. Since then, there has been extensive scale-up of treatment services: in 2008, an estimated 2.9 million people in the region were receiving ART, a 30-fold increase since in the end of 2003 [2]. Thus, early findings and initial hypotheses about the effects of HAART use on the proximate and underlying determinants of fertility may be incomplete, as they were indirectly predicated on the novelty and scarcity of HAART availability. In particular, an important feature inadequately considered in the conceptual model that guided this research is the failure to consider sufficiently the degree to which access to HIV treatment services would provide a primary point of regular access to health care, in a way that was not previously available to many women [41]. In addition, while the framework addressed the actual receipt or non-receipt of HAART at the individual level, other recent findings suggest that the availability and accessibility of HIV treatment services in a given community and the role of HAART optimism [46] may be additionally relevant variables. Thus, while the framework remains a useful tool for the development of hypotheses to guide future research regarding the impact of HAART on contraceptive use, our group is currently working to update it to include more recently available empirical findings.

Contraceptive method preference may have implications for both HIV transmission and unintended pregnancy risks and we found important differences in the types of contraceptive methods used by women in each of our three groups. While condoms are recommended to prevent HIV transmission to uninfected sexual partners, they are less effective than hormonal contraception and sterilization at preventing pregnancy [38]. Overall, a substantial proportion of women in this setting report relying exclusively on the male condom for preventing pregnancy (29%). An additional 25% of women report using condoms in conjunction with a hormonal/permanent method of contraception, resulting in over half of our sample reporting consistent condom use with or without another method. HAART users reported the highest prevalence of consistent condom use. Indeed much of the difference in contraceptive prevalence between our three groups was accounted for by the significantly lower prevalence of condom use among HIV-negative women. Only 39% of HIV-positive women reported consistent condom use (with our without another contraceptive method). While this prevalence is comparable to condom use rates reported in South Africa overall [41], all of the HIV-negative women in our study were currently sexually active, over 40% do not know the HIV-status of their primary partner, and over two-thirds desire (more) children. This observed pattern highlights the concerning risk environment for HIV acquisition among women of reproductive age in this hyper-endemic setting.

Unlike barrier methods, permanent and hormonal contraceptive methods are highly effective at preventing pregnancy but have no role in the prevention of HIV transmission [38]. Overall, 7% of women used permanent methods (hysterectomy and/or female sterilization) with small differences by HIV status and HAART use. The overall prevalence of sterilization is slightly lower than reported rates from South African women in general [41].

Compared with HAART-naïve women, HAART users were more likely to use hormonal contraception, and uptake of DMPA injectables exceeded oral contraceptive use. Reports from other settings suggest that DMPA use is rising owing to its discretion and convenient three-month dosing which corresponds with the HAART follow-up schedule [47]. Higher uptake of progesterone-only injectables in HAART users may also reflect provider preference based on concerns about possible interactions between HAART and estrogen-containing oral contraceptives [48]. Available guidelines advise that women receiving antiretroviral agents should use alternative or additional methods of contraception, beyond oral contraceptives [48].

There is no single method that can reliably assist HIV-positive women who wish to avoid pregnancy and HIV transmission to sero-discordant partners. Moreover, given concerns noted above about potential interaction between hormonal contraception and antiretroviral agents, dual protection is encouraged. In our study, not only were HAART users significantly more likely to use contraceptive methods overall, they were more likely than non-HAART users and HIV-negative women to use dual protection. Low prevalence of dual protection among HAART-naïve women (24%), with largely unsuppressed HIV viral load, reflects a population at risk of transmitting HIV to sero-discordant partners and, if they do become pregnant, are a population most requiring initiation of antiretroviral prophylaxis through PMTCT services.

Overall, 14% of HAART users, 18% of HAART-naïve women, and 31% of HIV-negative women were not using any form of contraceptive, suggesting risk for unintended pregnancy. A high proportion of women were also unaware of their partner's HIV status (42% overall). As such, the risks of conception-related HIV acquisition or transmission between sero-discordant couples are serious and integrated HIV and sexual and reproductive health services must be provided to help HIV-affected couples safely achieve their fertility goals.

Limitations of this study must be acknowledged. First, the cross-sectional nature of this analysis precludes us from determining causality between the explanatory variable and the outcome, particularly since contraceptive use, HIV status, and HAART use were assessed at the same point in time. Although reverse causality is considered unlikely (i.e., contraceptive use leading to HAART use), longitudinal studies are needed to investigate this relationship and would enable examination of changes in fertility intentions and contraceptive use over time. Second, there is a risk of social desirability bias whereby HIV-positive women may over-report their contraceptive use (and condom use, in particular) because of pressure from health workers and community members to practice protected sex [49], [50]. If over-reporting was differential, then our effect estimates are likely somewhat inflated. We took precautions against reporting bias by using standardized questions of contraceptive use and employing non-clinic staff to conduct the interviews. Third, there were important baseline differences between the HIV-positive and HIV-negative women in our study, a potential source of selection bias, which cannot be fully adjusted for in the analyses. In particular, HIV-positive women in our study were significantly older and age is a known predictor of contraceptive use and is associated with a number of other covariates (e.g., parity, education status). In an attempt to address this limitation, we conducted a sub-analysis of contraceptive use restricted to women less than 35 years of age. We found no differences in the variables that predicted contraceptive use, nor the magnitude of the associations. The results of the sub-analysis suggest that our overall findings are robust, despite differences in age at baseline. Finally, a quantitative analysis such as this fails to capture the salient influence of cultural and gender dynamics on contraceptive decision-making. Indeed, qualitative studies from this setting have highlighted the importance of considering the real and perceived side effects of contraceptives and partner influence and status as mitigating factors influencing contraceptive decision-making of HIV-positive women [4].

In conclusion, our results demonstrate that HIV-positive women overall and women accessing HAART services in particular, are more likely to use contraception overall, and more likely to use barrier, permanent, and dual protection methods in particular, compared with their HIV-negative and HAART-naïve counterparts. The contraceptive use profile of HIV-positive and HIV-negative women in Soweto highlights the need for improved integration of HIV testing, treatment, and care services with reproductive and sexual health services, including the provision of effective contraception. Through the prevention of unintended pregnancy, integrated services are likely to benefit maternal and child health, increase primary prevention of vertical transmission, and decrease incidence of conception-related horizontal transmission to discordant sexual partners [51].

## Supporting Information

Table S1 Adjusted sub-analyses of variables associated with contraceptive use among HIV-positive (HAART users and HAART-naïve) and HIV-negative women (aged 18–34 years, currently sexually active, and non-pregnant) in Soweto, South Africa (n = 420)
(0.01 MB DOCX)
