# Supplementary material for: Contraceptive Use and Method Preference among Women in Soweto, South Africa: The Influence of Expanding Access to HIV Care and Treatment Services
Source: PLoS One. 2010 Nov 5;5(11):e13868. doi: 10.1371/journal.pone.0013868 (PMC2974641; doi:10.1371/journal.pone.0013868)
Supplement: Table S1 — Adjusted sub-analyses of variables associated with contraceptive use among HIV-positive (HAART users and HAART-naïve) and HIV-negative women (aged 18–34 years, currently sexually active, and non-pregnant) in Soweto, South Africa (n = 420) (0.01 MB DOCX) [file pone.0013868.s001.docx]

**Supplemental Table 1: Adjusted sub-analyses of variables associated with contraceptive use among HIV-positive (HAART users and HAART-naïve) and HIV-negative women (aged 18-34 years, currently sexually active, and non-pregnant) in Soweto, South Africa (n=420)**

| **Variable** | **Adjusted Odds Ratio (AOR)** | |
| --- | --- | --- |
|  | **AOR** | **95% CI** |
| **HIV and HAART Use Status**  HIV-negative  HIV-positive, HAART-naïve  HIV-positive, receiving HAART | Ref.  1.27  2.24 | Ref.  0.65, 2.48  1.05, 4.99 |
| **Age** (per increase in year) | 0.98 | 0.92, 1.05 |
| **Education**  Less than Grade 12  Grade 12 or higher | Ref.  0.53 | Ref.  0.30, 0.91 |
| **Number of living children**  0  1  2+ | Ref.  0.81  2.31 | Ref.  0.44, 1.49  0.94, 5.69 |
| **Fertility Intentions**  Yes  No | Ref.  1.72 | Ref.  0.95, 3.12 |

Notes:

Ref. = Reference category

95% CI = 95% Confidence Interval
